# Therapeutic dilemma’s: antipsychotics use for neuropsychiatric symptoms of dementia, delirium and insomnia and risk of falling in older adults, a clinical review

**DOI:** 10.1007/s41999-023-00837-3

**Published:** 2023-07-26

**Authors:** Netta Korkatti-Puoskari, Miia Tiihonen, Maria Angeles Caballero-Mora, Eva Topinkova, Katarzyna Szczerbińska, Sirpa Hartikainen, on the Behalf of the EuGMS Task & Finish group on FRIDs

**Affiliations:** 1 School of Pharmacy, University of Eastern Finland, Kuopio, Finland; 2 Hospital General Universitario de Ciudad Real, Ciudad Real, Spain; 3 Geriatric Department, First Faculty of Medicine, Charles University, General Faculty Hospital, Prague and Faculty of Health and Social Sciences, South Bohemian University, Ceske Budejovice, Czech Republic; 4 Medical Faculty, Epidemiology and Preventive Medicine Chair, Laboratory for Research on Ageing Society, Jagiellonian University Medical College, Kraków, Poland

**Keywords:** Antipsychotics, Falls, Dementia, Delirium, Insomnia, Older adults

## Abstract

**Purpose:**

Because of the common and increasing use of antipsychotics in older adults, we aim to summarize the current knowledge on the causes of antipsychotic-related risk of falls in older adults. We also aim to provide information on the use of antipsychotics in dementia, delirium and insomnia, their adverse effects and an overview of the pharmacokinetic and pharmacodynamic mechanisms associated with antipsychotic use and falls. Finally, we aim to provide information to clinicians for weighing the benefits and harms of (de)prescribing.

**Methods:**

A literature search was executed in CINAHL, PubMed and Scopus in March 2022 to identify studies focusing on fall-related adverse effects of the antipsychotic use in older adults. We focused on the antipsychotic use for neuropsychiatric symptoms of dementia, insomnia, and delirium.

**Results:**

Antipsychotics increase the risk of falls through anticholinergic, orthostatic and extrapyramidal effects, sedation, and adverse effects on cardio- and cerebrovascular system. Practical resources and algorithms are available that guide and assist clinicians in deprescribing antipsychotics without current indication.

**Conclusions:**

Deprescribing of antipsychotics should be considered and encouraged in older people at risk of falling, especially when prescribed for neuropsychiatric symptoms of dementia, delirium or insomnia. If antipsychotics are still needed, we recommend that the benefits and harms of antipsychotic use should be reassessed within two to four weeks of prescription. If the use of antipsychotic causes more harm than benefit, the deprescribing process should be started.

**Supplementary Information:**

The online version contains supplementary material available at 10.1007/s41999-023-00837-3.

## Key summary points

**Purpose:**

This clinical review summarizes the current evidence on the risks of antipsychotic-related falls in older adults, including aspects of pharmacokinetics and -dynamics to assist clinicians in (de)prescribing antipsychotics in older people.

**Findings:**

Antipsychotics are widely used for neuropsychiatric symptoms of dementia, delirium and insomnia in older adults despite off-label indications. Antipsychotics increase the risk of falls due to anticholinergic and extrapyramidal effects, sedation, and cardio- and cerebrovascular adverse effects.

**Message:**

Consider deprescribing of antipsychotics in older adults at risk of or with falls if there is no current indication, or if safer alternatives are available, or if antipsychotics are prescribed for neuropsychiatric symptoms of dementia, insomnia, or delirium.

**Supplementary Information:**

The online version contains supplementary material available at 10.1007/s41999-023-00837-3.

## Introduction

Antipsychotics have been in use for about 70 years for the management of psychotic disorders, mainly schizophrenia [1]. The first typical antipsychotics developed, such as levomepromazine and later haloperidol, mainly block dopamine D_2 _receptors [1, 2]. Newer atypical antipsychotics like risperidone may also block dopamine D_1_ and D_4_ and serotonin 5-HT_2_ receptors. According to a recent systematic review and meta-analysis antipsychotics increase the risk of falling by about 50% in older adults [3].

The use of antipsychotics in the USA varies by age groups, reaching 1.5% in adults between 60–64 years old and rising to 2.1% in those aged 80–84 years [4]. In European countries, the use of antipsychotics in home-care patients varies from 3.0 to 12.4% [5], rising to 26.4% in the nursing home setting [6] and to 35.6% if only considering residents with severe cognitive impairment [7]. According to the recent Canadian Institute for Health Information report, 22% of residents in long-term care homes were treated with antipsychotics without a proper indication [8].

The most common (off-label) indications for antipsychotic use among older adults, especially those with cognitive decline, are neuropsychiatric symptoms of dementia and delirium. Over 55 million people –mainly older adults– are diagnosed with dementia globally [9]. More than 90% of persons with cognitive disorders experience neuropsychiatric symptoms at least at some point during their disease process [10]. The three most common neuropsychiatric symptoms in people with cognitive impairment initiating antipsychotics were agitation/aggressiveness (27%), irritability (23%) and depression (16%) [11]. Antipsychotics are commonly given for the treatment of neuropsychiatric symptoms in older people with dementia despite the known potential risk of adverse events and mainly with off-label indications [12]. Antipsychotic use is common in community-dwelling people with Alzheimer’s disease (AD) (24%) [13], rising 2–3 years before formal diagnosis [14]. Risperidone (54%), quetiapine (30%) and haloperidol (6%) were the three most frequently used long-term antipsychotics in people with AD [15]. Dementia itself is associated with a higher rate of falling [16], and incident hip fracture risk is over two-fold in the AD population [17].

Delirium is a serious health condition, and its prevalence varies from 7 to 35% in older adults in the emergency department [18] to 49% in hospitalized older people with dementia in intensive care units and surgical departments [19]. Delirium increases the risk of falling [20] and other serious outcomes, and it is also associated with an increased risk of death and institutionalization [21]. Symptoms of delirium have been commonly treated with antipsychotics in a variety of care settings [22–24]. However, according to a 2016 systematic review and meta-analysis, nor was it associated with changes in duration or severity of delirium, length of hospital or intensive care unit stay, or mortality [24].

Nguyen et al. summarize in their review that the prevalence of insomnia symptoms among older adults reaches up to 75% [25]. Insomnia increases the risk of falling [26]. Of people with dementia or cognitive impairment, 60% to 70% have sleep disturbances [27]. Antipsychotics are not a first-line treatment for sleeping problems, but these drugs are used for insomnia [28]. In a Canadian population-based retrospective study focused on community-dwelling older adults with sleep disorders, it was found that 2.4% of the subjects included received antipsychotics, such as quetiapine (88%) or risperidone (9%) [28].

In this paper, we conducted a literature search focusing on the clinical dilemma of deprescribing antipsychotics in older people at risk of falling. We aim to provide an overview of the current knowledge on the causes for antipsychotic-related risk of falling in older adults. We aim to summarize the benefits and adverse effects for specific indications focusing on neuropsychiatric symptoms of dementia, delirium, or insomnia. In addition, we collected information on pharmacokinetics and pharmacodynamics related to the risks of falling due to antipsychotics as well as information to assist clinicians to consider the benefits and harms of (de)prescribing antipsychotics.

## Search strategy

The literature search was conducted in March 2022 via CINAHL, PubMed and Scopus. The exact search terms can be found in Supplementary Table 1. Studies that focused on the reasons behind the antipsychotic-related fall risk in older adults were searched for. Included studies needed to focus on delirium, dementia and insomnia and were written in English language. Studies that focused on psychosis, schizophrenia, and psychotic symptoms, depression, mania and bipolar disorder, intractable hiccups, and post-traumatic stress disorder were excluded, unless these conditions were related to neuropsychiatric symptoms of dementia, delirium, or insomnia. Studies focusing on lithium were also excluded. Studies were included if the age of the patients was 65 years or older or the mean age was 70 years or older. Only original articles, reviews, systematic reviews, and meta-analyses were included. There were no time limits for the studies. Book chapters, case reports, conference papers, congress abstracts, editorials, letters, notes, and posters were excluded. We selected all discovered studies based on title, abstract and furthermore full text to meet the aims of this study. A total of 1,287 studies were identified, and 943 studies remained after removal of 344 duplicates. Finally, 540 studies were identified as meeting the aims after title screening and furthermore 52 studies after abstract and full text screening. In addition, we included articles based on additional literature searches of papers listed in the references to find more information.

## Medication review and reconciliation

It is recommended that older adults are assessed annually for fall risk, and a medication review is suggested annually for all older adults and every six months for frail or vulnerable adults [29, 30]. A medication review is based on a comprehensive geriatric assessment or a holistic examination done by a physician or an interdisciplinary team [31]. In medication review, it is important to check that all medications including antipsychotics have a current and appropriate indication and dose for their use.

### Matching antipsychotic use to an appropriate indication

According to the Food and Drug Administration (FDA), the indications for antipsychotics in adults are psychotic disorders, schizophrenia, schizoaffective disorder, treatment-resistant depression, major depressive disorder, bipolar disorder, agitation, generalized nonpsychotic anxiety, severe behavioural problems, hyperactivity and Tourette’s syndrome [32]. Indications vary between different drug substances. In Europe, only risperidone has an official indication for persistent aggression in patients with moderate or severe dementia of AD [33]. The use of other antipsychotics for neuropsychiatric symptoms of dementia and all antipsychotic use for delirium and insomnia is therefore off-label use.

Although antipsychotics are commonly prescribed for delirium, the current evidence does not support this practice [24]. A 2016 systematic review and meta-analysis, which included 19 studies and focused on the use of antipsychotics for the prevention and treatment of delirium in hospitalized adults found that antipsychotic use was not associated with changes in the duration or severity of delirium. As well, when considering seven studies that compared antipsychotics with placebo or no treatment for the prevention of delirium after surgery, there was no significant effect on delirium incidence [24]. Although there are conflicting studies on the usefulness of antipsychotics in the treatment of delirium [34], the first-line treatment of delirium is non-pharmacological interventions and identification and treatment of the possible underlying cause of the delirium [35]. Medications that increase the risk of delirium should be reviewed, and if possible, and deprescribed.

Atypical antipsychotics are not recommended for insomnia in older adults [36]. Although some antipsychotics, such as quetiapine and olanzapine, have sedative effects via their antagonism of the histamine H1 receptor [37], there is limited evidence on the use of antipsychotics for insomnia in older adults and their use is not recommended for the treatment of insomnia [36–38]. The first-line treatment for insomnia is an effective cognitive behavioural therapy [39], in addition to deprescribing drugs that may cause insomnia and treating chronic diseases and symptoms such as pain as well as possible to maintain sleep.

The first-line management of neuropsychiatric symptoms of dementia is to identify by comprehensive clinical examination possible diseases, triggers, or other causes for these neuropsychiatric symptoms and then treatment should focus on the causes [40]. Antipsychotics are often used for neuropsychiatric symptoms, although non-pharmacological treatment options such as psychosocial interventions are first-line treatments [11, 12, 40]. Antipsychotics should be considered as a last resort when other treatment options have not been successful, in severe agitation, or when the risk of harm to self or others outweighs the potential secondary effects of the medication used [33, 40]. If antipsychotics are needed, their use should be limited to the shortest possible duration and lowest effective dose [40].

Commonly used international guidelines, such as the European Screening Tool of Older Persons’ Prescriptions (STOPP) and the North American Beers Criteria for Potentially Inappropriate Medication Use in Older Adults, recommend avoiding antipsychotics in older adults with neuropsychiatric symptoms of dementia unless the symptoms are severe and other non-pharmacological treatment options have failed [41, 42]. The Beers Criteria also include delirium and advice not to prescribe antipsychotics unless the older adult is at the risk of significant harm to self or others [42]. If there is a risk of harm, both The National Institute for Health and Care Excellence and The American Geriatrics Society recommend that haloperidol for delirium can be used for a short duration, e.g., for one week or less [43, 44]. The Screening Tool of Older Persons Prescriptions in older adults with high fall risk (STOPPFall) also recommends considering withdrawal of antipsychotic if they are prescribed for insomnia or for neuropsychiatric symptoms of dementia [45]. Usually, the doses of antipsychotics for neuropsychiatric symptoms of dementia, delirium and insomnia are just a minor part (such as 0.5–1.0 mg of risperidone /day) of the dose recommended for the treatment of psychosis, nevertheless the risk of adverse drug events remains high in older people, even at lower doses [24, 37, 40].

### Pharmacodynamics, pharmacogenetics and drug–drug interactions

The main metabolic routes of the most used antipsychotics are described in Table 1. Additional prescribing recommendations for different types of metabolizers are also listed. Additional prescribing recommendations are given for risperidone, quetiapine, clozapine, aripiprazole, and haloperidol. There are recommendations for CYP2D6 poor and/or ultrarapid metabolizers and one for CYP3A4 poor metabolizers. In Europe, 1.2% to 12% are CYP2D6 poor metabolizers and up to 9.1% are CYP2D6 ultrarapid metabolizers [46]. The main drug-drug interactions of the most used antipsychotics in older adults are also described in Table 1.

**Table 1:** The pharmacokinetics, pharmacogenetics, and drug-drug interactions of antipsychotics

Antipsychotics	Main metabolic pathway _47_	Additional prescription recommendations regarding different metabolizer types _48_	Drug-drug interaction/Pharmacokinetic interaction with Antipsychotics _47_
Atypical antipsychotics			
Risperidone	CYP2D6-mediated metabolism Active metabolite (9-hydroxyrisperidone = paliperidone) CYP3A4 mediate metabolism	Dutch Pharmacogenetics Working Group (DPWG): Dose reduction for CYP2D6 poor metabolizers. For CYP2D6 ultrarapid metabolizers: Alternative drug	CYP3A4 inducers like carbamazepine reduce the plasma concentrations of risperidone CYP2D6 inhibitors like fluoxetine and paroxetine increase the plasma concentrations of risperidone
Paliperidone	CYP2D6-mediated metabolism CYP3A4-mediated metabolism (limited role)	No recommendations	P-glycoprotein substrate. Carbamazepine can strongly induce both CYP3A4 and p-glycoprotein and decrease the plasma concentrations of paliperidone
Quetiapine	CYP3A4-mediated metabolism One of three metabolites is active metabolite (N-desalkyl quetiapine) Metabolism is reduced by ca 30% with ageing Similar mechanism of action via antagonism of histamine H_1_ receptor with mirtazapine and low-dose doxepin _39_	DPWG: Other indications than depression: Dose reduction for CYP3A4 poor metabolizers	CYP3A4 inducers like carbamazepine and St John’s wort will increase quetiapine clearance CYP3A4 inhibitors like ketoconazole may increase quetiapine plasma concentrations
Olanzapine	UGT1A4-mediated direct glucuronidation CYP1A2-mediated metabolism CYP2D6-mediated oxidation 40% of dose removed via first-pass metabolism Similar mechanism of action via antagonism of histamine H_1_ receptor with mirtazapine and low-dose doxepin _39_	No recommendations	Fluvoxamine increases the plasma concentrations of olanzapine. Smoking increases the olanzapine clearance by 40%. In females, olanzapine clearance is decreased by 30%
Aripiprazole	CYP2D6-mediated metabolism CYP3A4-catalyzed dehydrogenation Active metabolite dehydroaripiprazole	DPWG: Dose reduction for CYP2D6 poor metabolizers	CYP3A4 inducers like carbamazepine and ketoconazole lower aripiprazole plasma concentrations by ca 60% CYP3A4 inhibitors like itraconazole increase the plasma concentrations by ca 45%
Clozapine	Extensive first-pass metabolism in the liver and gut CYP1A2-mediated metabolism CYP2D6-mediated metabolism CYP3A4 (minor)-mediated metabolism, to active norclozapine	No recommendations. FDA _49_: Dose reduction for CYP2D6 poor metabolizers	CYP1A2 substrate caffeine may increase clozapine levels significantly (26%). Fluoxetine increases plasma concentrations of clozapine and norclozapine, possibly by CYP1A2 inhibition Sertraline may significantly increase the plasma concentrations of clozapine
Typical antipsychotics			
Haloperidol	Glucuronidation (main route) CYP3A4-mediated reduction (minor role) CYP2D6-mediated reduction (minor role) _48_	DPWG: Dose reduction for CYP2D6 poor metabolizer Alternative drug for CYP2D6 ultrarapid metabolizers	Is a CYP3A4 and CYP2D6 substrate and inhibitor. Reduced form is also a CYP3A4 substrate and a CYP2D6 inhibitor. Interactions with most of the drugs not clinically significant. Co-administration with carbamazepine may clinically significantly change the pharmacokinetics of haloperidol
Chlorpromazine	CYP2D6-mediated metabolism (major role) CYP1A2-mediated metabolism (minor role)	No recommendations	CYP1A2 inhibitors like ciprofloxacin and fluvoxamine can increase the plasma concentrations of chlorpromazine. CYP2D6 inhibitors like fluoxetine and paroxetine can increase the plasma concentrations of chlorpromazine
Levomepromazine	No data	No recommendations	No data

## Fall-related adverse effects of antipsychotics

Antipsychotics increase the risk of falling [3]. According to a recent Delphi study by the European Geriatric Medicine Society Task and Finish Group on Fall-Risk-Increasing Drugs, the fall risk associated with antipsychotics is caused by several mechanisms such as their anticholinergic, sedative and extrapyramidal effects, as well as their cardio- and cerebrovascular effects and orthostatic hypotension. The prevalence of fall-related adverse effects of antipsychotics is shown in Table 2. The information is based on summaries of product characteristics.

**Table 2:** Prevalence of fall-related adverse effects of antipsychotics based on summaries of product characteristics (Finnish Medicine Agency)

Antipsychotics	Cardiovascular events (e.g., prolonged QT time)	(Orthostatic) hypotension	Sedation	Drowsiness or somnolence	Delirium or confusional stage	Dizziness or vertigo	Extrapyramidal symptoms (e.g., tardive dyskinesia, akathisia)	Confusion	Anticholinergic effects (e.g., blurred vision, urinary retention, constipation)
Atypical antipsychotics									
Risperidone (tablet)	+++	++	++++	++++	++	+++	++++	No data	+++
Paliperidone (Prolonged-release suspension for injection)	+++	++	+++	+++	+	+++	+++	No data	+++
Quetiapine (tablet)	+++	+++	No data	++++	++	++++	++++	No data	++++
Olanzapine (tablet)	++	++++	No data	++++	No data	+++	+++	No data	+++
Clozapine (tablet)	++++	+++	++++	++++	+	++++	+++	+	++++
Aripiprazole (tablet)	++	++	+++	+++	No data	+++	+++	No data	+++
Brexpiprazole (tablet)	Unknown	++	+++	No data	No data	+++	+++	No data	No data
Ziprasidone (capsule)	+++	++	+++	++++	No data	+++	+++	No data	+++
Lurasidone (tablet)	+++	++	+++	+++	No data	+++	++++	No data	+++
Cariprazine (capsule)	+++	++	+++	No data	++	+++	++++	No data	+++
Asenapine (Sublingual tablet)	++	++	+++	++++	No data	+++	+++	No data	+
Amisulpride (tablet)	++	+++	No data	+++	No data	No data	++++	++	+++
Typical antipsychotics									
Haloperidol (tablet)	++	+++	++	+++	++	+++	++++	No data	+++
Chlorpromazine (tablet)	+++	++++	++++	++++	No data	No data	++++	No data	++++
Levomepromazine (tablet)	+	++++	++++	++++	No data	++++	++++	+	++
Promazine (tablet)	No data	No data	No data	No data	No data	No data	No data	No data	No data
Perphenazine (tablet)	+	+++	+++	+++	No data	No data	+++	++	++
Prochlorperazine (tablet)	++++	++++	No data	++++	No data	No data	++++	No data	++++
Trifluoperazine (tablet)	No data	No data	No data	No data	No data	No data	No data	No data	No data
Periciazin (capsule)	No data	No data	No data	No data	No data	No data	No data	No data	No data
Thioridazine (tablet)	No data	No data	No data	No data	No data	No data	No data	No data	No data
Droperidol (injection)	++	+++	No data	+++	+	++	+++	No data	No data
Flupentixol (tablet)	No data	++	No data	+++	No data	+++	++++	++	+++
Chlorprothixene (tablet)	++++	++++	No data	++++	No data	++++	+++	No data	+++
Zuclopenthixol (tablet)	+++	+++	No data	++++	No data	++++	++++	+++	++++
Pimozide (tablet)	No data	No data	No data	No data	No data	No data	No data	No data	No data
Sulpiride (capsule)	+	++	+++	+++	No data	No data	+++	Unknown	+++
Melperone (tablet)	No data	No data	No data	No data	No data	No data	No data	No data	No data
Tiapride (tablet)	No data	No data	No data	No data	No data	No data	No data	No data	No data

++++:  > 1/10 (very common ≥ 1/10)

+++: 1/10–1/100 (common ≥ 1/100 to < 1/10)

++: 1/100–1/1000 (uncommon: ≥ 1/1000 to < 1/100)

+: < 1/1000 (Rare: ≥ 1/10,000 to < 1/1000) and (very rare < 1/10,000)

Not known: cannot be estimated from the available data

### Sedation and anticholinergic effects

Antipsychotics have sedative effects [Table 2, 50, 51] through several mechanisms [2, 37]: antagonism of histamine H_1_ receptors, muscarinic receptors and adrenergic α_1_-receptors. The typical antipsychotics haloperidol and chlorpromazine act as antagonists on all three receptors [2]. The atypical antipsychotics quetiapine and olanzapine cause sedation by antagonising the histamine H_1_ receptor [39]. Quetiapine is also a weak antagonist of adrenergic α_1_-receptors, and olanzapine is an antagonist of both muscarinic and adrenergic α_1_-receptors [2]. A network meta-analysis comparing the safety and efficacy of atypical antipsychotics in the treatment of neuropsychiatric symptoms of dementia found no differences in sedative effects between aripiprazole, olanzapine and quetiapine. However, risperidone was found to be less sedating than quetiapine and olanzapine [50]. This has also been reported elsewhere [51].

Antipsychotics have anticholinergic adverse effects, both central (worsening of cognition, delirogenous potential) and peripheral, such as blurred vision, dry mouth, urinary retention or constipation, due to their antagonism of muscarinic receptors [Table 2, 51]. Both typical and some atypical antipsychotics such as olanzapine and clozapine are associated with antagonism of muscarinic receptors [2]. However, the exact mechanism underlying the anticholinergic adverse effects of some atypical antipsychotics like risperidone and quetiapine, is still unclear.

### Cardio- and cerebrovascular adverse outcomes

The FDA required a boxed warning for atypical antipsychotics in 2005, partially due to the death-risk increasing cardiovascular adverse outcomes, e.g., heart failure in people with dementia-related psychosis [52]. In 2008, the FDA extended the boxed warning requirement were extended to typical antipsychotics and the EMA made a similar recommendation [53, 54].

The use of typical [55–57] and atypical antipsychotics [55–58] is associated with serious cardio- and cerebrovascular adverse events, such as stroke and myocardial infarction and higher mortality. Reasons for these cardio- and cerebrovascular adverse effects include affinity to multiple receptors as described at the beginning of Sect. "Fall-Related Adverse Effects of Antipsychotics" as well as an adverse event profile including orthostatic hypotension, QTc prolongation, tachycardia, and metabolic effects. In a Welsh cohort study people with dementia receiving typical and atypical antipsychotics had a higher risk of having stroke and venous thromboembolism compared to nonusers of antipsychotics [59]. A recent Finnish register-based cohort study among people with AD who were new users of antipsychotic and matched with nonusers showed a different result [55]. There was no overall association between antipsychotic use and the risk of stroke for longer durations of antipsychotic use compared with non-use. However, during the first 60 days of use, antipsychotic initiators had a higher age-standardized incidence rate of stroke and an increased risk of stroke, as well for ischaemic stroke. In this study, risperidone (63%) and quetiapine (30%) were the most commonly used antipsychotics and there was no difference between these drugs in the risk of any stroke or ischaemic stroke. A previous Korean cohort study compared the risk of ischaemic stroke between older people taking risperidone and haloperidol and reported higher a incidence rate of ischaemic stroke among haloperidol users (6.43 /1000 person-years) than in the risperidone users (2.88/1000 person-years) [60].

Antipsychotics can cause QT prolongation by acting on cardiac ion channels [61]. If the QT time is more than 500 ms, there is a high risk of serious adverse events such as torsade de pointes and ventricular arrythmias [62]. Ziprasidone and thioridazine have a particularly high risk of prolonging QTc, quetiapine and chlorpromazine have an intermediate risk, but risperidone, clozapine, olanzapine, aripiprazole, and haloperidol have a low risk of prolonging QTc.

Antipsychotics cause orthostatic hypotension via α_1_-adrenoceptor blockade [63]. Blocking of α_1_-adrenoceptors attenuates the increase in systemic vascular resistance, a response to postural change, leading to symptoms of orthostatic hypotension and tachycardia. Among the typical antipsychotics, quetiapine, olanzapine, clozapine, and amisulpride have potent orthostatic hypotensive effect (Table 2). Among typical antipsychotics, a strong (orthostatic) hypotensive effect can be found with haloperidol, chlorpromazine, levomepromazine, perphenazine, droperidol, prochlorperazine, chlorprothixene, and zuclopenthixol.

### Extrapyramidal effects

Both typical and atypical antipsychotics can cause extrapyramidal effects [Table 2, 50, 51, 61, 64]. The typical antipsychotics haloperidol and chlorpromazine in particular cause extrapyramidal effects [61]. The situation is less clear for atypical antipsychotics. Yunusa and colleagues conducted a network meta-analysis of randomized clinical trials comparing atypical antipsychotics (aripiprazole, olanzapine, quetiapine, and risperidone) with placebo or head-to-head comparisons of at least two atypical antipsychotics in older adults with neuropsychiatric symptoms of dementia [50]. They found that risperidone was associated with an increased risk of extrapyramidal symptoms when compared with quetiapine (OR 3.75; 95% CI 1.61–8.73), whereas quetiapine had a lower risk compared to olanzapine (OR 0.39; 95% CI 0.16–0.93).

## Fall-related injuries associated with antipsychotics use

Antipsychotics are associated with fall related injuries like hip fractures and head injuries as adverse effects [65–67]. According to a systematic review and meta-analysis, antipsychotics increase the risk of fragility hip fractures with OR 1.85 (95% CI 1.36–2.51) in the general older population [65]. Koponen et al. (2017) aimed to investigate association of antipsychotic use with the risk of hip fracture in persons with AD and to compare the risk according to the duration of use and use of quetiapine or risperidone [66]. The risk of hip fracture was about the same compared to nonusers of antipsychotics among older adults with AD (aHR 1.54; 95% CI 1.39–1.70). The risk increased from the first days of use and remained elevated thereafter and the risk of hip fracture was about the same for risperidone and quetiapine users. In addition, falls can cause head injury and traumatic brain injury. In older adults with AD the antipsychotic use was associated with an increased event rate for traumatic brain injuries and the risk was higher in quetiapine users than in risperidone users [67].

## Deprescribing antipsychotics to reduce fall risk 

Deprescribing of antipsychotics should always be considered if there is no current indication or if there is a safer alternative available [43]. In addition, antipsychotic withdrawal should be considered when antipsychotics are prescribed for neuropsychiatric symptoms of dementia or for sleep disorders, and withdrawal should be considered when antipsychotics are prescribed for bipolar disorder. Deprescription should especially be considered if extrapyramidal or cardiac adverse effects, dizziness, (signs of) sedation, or blurred vision occur with antipsychotic use.

### Taper and timing of deprescribing

STOPPFall, MedStopper and Deprescribing.org are some examples of tools to assist clinicians in deprescribing medication [43, 70, 71]. The STOPPFall deprescribing tool was developed by the European Geriatric Medicine Society (EuGMS) Task and Finish Group on FRIDs (fall-risk increasing drugs) in collaboration with the EuGMS Special Interest Group on Pharmacology through a European expert Delphi consensus effort [43]. The expert group has developed a decision tree for antipsychotic withdrawal [43, 72]. It focuses on the prevention of falls and includes algorithms for practical deprescribing [72]. There is also a description of deprescribing process by Scott et al. [73].

The panellists contributing to STOPPFall strongly agree that stepwise withdrawal is generally needed when deprescribing antipsychotics [43]. The deprescribing process depends on the indication for which antipsychotics are being given for and on the duration of the treatment [72]. For example, if antipsychotics are being given for neuropsychiatric symptoms of dementia for at least three months, it is recommended that the dose be reduced by 25–50% every 1–2 weeks. If antipsychotics are given for insomnia and the likely underlying comorbidities are treated, the antipsychotic can be stopped without tapering. The MedStopper [70] recommends a 25% dose reduction each week if antipsychotics are used daily for more than 3–4 weeks. The tapering can be extended or decreased for example to a 10% dose reduction, if necessary. However, individual countries may differ in their recommendations for the timing of medication reviews after prescribing antipsychotics, e.g. in the USA tapering and stopping of antipsychotics is recommended within 4 weeks if there is no clinically relevant response and within 4 months if the medication has been shown to be effective in reducing symptoms, and in Australia reassessment of the need for antipsychotic treatment is recommended within 4 to 12 weeks [68, 69]. Several studies have suggested that the time frame for reassessing should be shorter than in these clinical practice guidelines [55].

A few studies have examined the outcomes of deprescribing antipsychotics. Nursing home staff reported several benefits and barriers to reducing antipsychotics medication [74]. Benefits reported included improvements in the quality of life, family satisfaction and reductions in falls and injuries. Barriers included return or worsening of symptoms, lack of effectiveness and/or lack of staff resources for non-pharmacological management strategies and family resistance.

### Monitoring during and after deprescribing

After deprescribing, stopping or dose reduction, it is recommended to monitor for changes in symptoms e.g., orthostatic hypotension, dizziness, fall incidents and recurrence of symptoms including aggression, agitation, delusion, hallucination, and psychosis [43, 72]. It is also recommended to consider monitoring of insomnia. Furthermore, follow-ups are advised to be organized based on an individual basis, e.g. according to the severity of neuropsychiatric symptoms or insomnia at the baseline and possible withdrawal symptoms [72].

## Conclusions

Antipsychotics are used for neuropsychiatric symptoms of dementia, delirium and insomnia in older adults despite the lack of proven efficacy and official indications. Neuropsychiatric symptoms should be assessed, and the causes should be thoroughly identified and treated. If symptoms persist after non-pharmacological interventions the syndromes could be treated with pharmacological treatments. Antipsychotics may be used only if other treatments have not been successful, or if the patient is severely agitated or at the risk of harm to self or others [10]. If antipsychotics are needed, they should be started and used at the lowest effective dose and used for as short a time as possible. It is important to remember that neuropsychiatric syndromes, delirium, and insomnia increase the risk of falling regardless of pharmacological treatment. In addition, antipsychotics increase the risk of falling through anticholinergic and extrapyramidal effects, sedation, (orthostatic) hypotension, and severe cardio- and cerebrovascular adverse effects**.** In addition, drug-drug interactions may increase the risk of falling because of possible increased exposure to antipsychotics and adverse effects.

The European STOPPFall tool recommends deprescribing antipsychotics if they have no indication, if they are prescribed for insomnia or neuropsychiatric symptoms of dementia, are ineffective or have severe adverse drug related effects. The deprescribing should also be considered if the patient experiences cardiac or extrapyramidal side effects, sedation, signs of sedation, blurred vision or dizziness. STOPPFall recommends that stepwise withdrawal is generally needed when deprescribing antipsychotics. When deprescribing antipsychotics, it is recommended to monitor for recurrence of symptoms such as aggression, agitation, delusions, hallucinations and psychosis, and to consider monitoring for insomnia.

## Supplementary Information

Below is the link to the electronic supplementary material.

Supplementary file1 (DOCX 18 KB)
