# Supplementary material for: Therapeutic dilemma’s: antipsychotics use for neuropsychiatric symptoms of dementia, delirium and insomnia and risk of falling in older adults, a clinical review
Source: Eur Geriatr Med. 2023 Jul 26;14(4):709–20. doi: 10.1007/s41999-023-00837-3 (PMC10447285; doi:10.1007/s41999-023-00837-3)
Supplement: Supplementary file 1 — Supplementary file1 (DOCX 18 KB) [file 41999_2023_837_MOESM1_ESM.docx]

**Supplementary material**

Supplementary table 1. Search terms

| Search terms |
| --- |
| PubMed, March 2022    #1 "Antipsychotic Agents"[mh] OR antipsychotic*[tw] OR "anti psychotic*"[tw] OR tranquil*[tw] OR neuroleptic*[tw] OR chlorpromazine[tw] OR levomepromazine[tw] OR promazine[tw] OR acepromazine[tw] OR triflupromazine[tw] OR cyamemazine[tw] OR chlorproethazine[tw] OR dixyrazine[tw] OR fluphenazine[tw] OR perphenazine[tw] OR prochlorperazine[tw] OR thiopropazate [tw] OR trifluoperazine[tw] OR acetophenazine[tw] OR thioproperazine[tw] OR butaperazine[tw] OR perazine[tw] OR periciazine[tw] OR thioridazine[tw] OR mesoridazine[tw] OR pipotiazine[tw] OR haloperidol[tw] OR trifluperidol[tw] OR melperone[tw] OR moperone[tw] OR pipamperone[tw] OR bromperidol[tw] OR benperidol[tw] OR droperidol[tw] OR fluanisone[tw] OR lumateperone[tw] OR oxypertine[tw] OR molindone[tw] OR sertindole[tw] OR ziprasidone[tw] OR lurasidone[tw] OR flupentixol[tw] OR clopenthixol[tw] OR chlorprothixene[tw] OR tiotixene[tw] OR zuclopenthixol[tw] OR fluspirilene[tw] OR pimozide[tw] OR penfluridol[tw] OR loxapine[tw] OR clozapine[tw] OR olanzapine[tw] OR quetiapine[tw] OR asenapine[tw] OR clotiapine[tw] OR sulpiride[tw] OR sultopride[tw] OR tiapride[tw] OR remoxipride[tw] OR amisulpride[tw] OR veralipride[tw] OR levosulpiride[tw] OR prothipendyl[tw] OR risperidone[tw] OR mosapramine[tw] OR zotepine[tw] OR aripiprazole[tw] OR paliperidone[tw] OR iloperidone[tw] OR cariprazine[tw] OR brexpiprazole[tw] OR pimavanserin[tw]  #2 Dementia[mh] OR Delirium[mh] OR "Sleep Initiation and Maintenance Disorders"[mh] OR Dyssomnias[mh] OR dement*[tw] OR delirium[tw] OR insomnia*[tw] OR dyssomnia*[tw] OR sleepless*[tw] OR "sleep disorder*"[tw] OR "sleep disturbance*"[tw] OR "sleep problem*"[tw] OR "sleep deprivation"[tw] OR "inadequate sleep"[tw] OR "insufficient sleep"[tw] OR "sleep insufficiency"[tw] OR "sleep debt"[tw]  #3 "Accidental Falls"[mh] OR fall[tw] OR falls[tw] OR falling[tw] OR fell[tw] OR slip*[tw] OR trip*[tw]  #4 Aged[mh] OR aged[tw] OR aging[tw] OR elder*[tw] OR older[tw] OR "old person*"[tw] OR "old people"[tw] OR "old man"[tw] OR "old men"[tw] OR "old woman"[tw] OR "old women"[tw] OR "old citizen*"[tw] OR "old resident*"[tw] OR "old inhabitant*"[tw] OR senior*[tw] OR retired[tw] OR retiree*[tw] OR pension*[tw]  #5 #1 AND #2 AND #3 AND #4  Results: 264 |
| Scopus, March 2022    #1 TITLE-ABS-KEY(antipsychotic* OR "anti psychotic*" OR tranquil* OR neuroleptic* OR chlorpromazine OR levomepromazine OR promazine OR acepromazine OR triflupromazine OR cyamemazine OR chlorproethazine OR dixyrazine OR fluphenazine OR perphenazine OR prochlorperazine OR thiopropazate OR trifluoperazine OR acetophenazine OR thioproperazine OR butaperazine OR perazine OR periciazine OR thioridazine OR mesoridazine OR pipotiazine OR haloperidol OR trifluperidol OR melperone OR moperone OR pipamperone OR bromperidol OR benperidol OR droperidol OR fluanisone OR lumateperone OR oxypertine OR molindone OR sertindole OR ziprasidone OR lurasidone OR flupentixol OR clopenthixol OR chlorprothixene OR tiotixene OR zuclopenthixol OR fluspirilene OR pimozide OR penfluridol OR loxapine OR clozapine OR olanzapine OR quetiapine OR asenapine OR clotiapine OR sulpiride OR sultopride OR tiapride OR remoxipride OR amisulpride OR veralipride OR levosulpiride OR prothipendyl OR risperidone OR mosapramine OR zotepine OR aripiprazole OR paliperidone OR iloperidone OR cariprazine OR brexpiprazole OR pimavanserin)  #2 TITLE-ABS-KEY(dement* OR delirium OR insomnia* OR dyssomnia* OR sleepless* OR "sleep disorder*" OR "sleep disturbance*" OR "sleep problem*" OR "sleep deprivation" OR "inadequate sleep" OR "insufficient sleep" OR "sleep insufficiency" OR "sleep debt")  #3 TITLE-ABS-KEY(fall OR falls OR falling OR fell OR slip* OR trip*)  #4 TITLE-ABS-KEY(aged OR aging OR elder* OR older OR "old person*" OR "old people" OR "old man" OR "old men" OR "old woman" OR "old women" OR "old citizen*" OR "old resident*" OR "old inhabitant*" OR senior* OR retired OR retiree* OR pension*)  #5 ( LIMIT-TO ( DOCTYPE,”ar” ) OR LIMIT-TO ( DOCTYPE,”re” ) OR LIMIT-TO ( DOCTYPE,”sh” ) )  #6 #1 AND #2 AND #3 AND #4 AND #5  Results: 877 |
| CINAHL, March 2022    #1 antipsychotic* OR "anti psychotic*" OR tranquil* OR neuroleptic* OR chlorpromazine OR levomepromazine OR promazine OR acepromazine OR triflupromazine OR cyamemazine OR chlorproethazine OR dixyrazine OR fluphenazine OR perphenazine OR prochlorperazine OR thiopropazate OR trifluoperazine OR acetophenazine OR thioproperazine OR butaperazine OR perazine OR periciazine OR thioridazine OR mesoridazine OR pipotiazine OR haloperidol OR trifluperidol OR melperone OR moperone OR pipamperone OR bromperidol OR benperidol OR droperidol OR fluanisone OR lumateperone OR oxypertine OR molindone OR sertindole OR ziprasidone OR lurasidone OR flupentixol OR clopenthixol OR chlorprothixene OR tiotixene OR zuclopenthixol OR fluspirilene OR pimozide OR penfluridol OR loxapine OR clozapine OR olanzapine OR quetiapine OR asenapine OR clotiapine OR sulpiride OR sultopride OR tiapride OR remoxipride OR amisulpride OR veralipride OR levosulpiride OR prothipendyl OR risperidone OR mosapramine OR zotepine OR aripiprazole OR paliperidone OR iloperidone OR cariprazine OR brexpiprazole OR pimavanserin  #2 dement* OR delirium OR insomnia* OR dyssomnia* OR sleepless* OR "sleep disorder*" OR "sleep disturbance*" OR "sleep problem*" OR "sleep deprivation" OR "inadequate sleep" OR "insufficient sleep" OR "sleep insufficiency" OR "sleep debt"  #3 fall OR falls OR falling OR fell OR slip* OR trip*  #4 aged OR aging OR elder* OR older OR "old person*" OR "old people" OR "old man" OR "old men" OR "old woman" OR "old women" OR "old citizen*" OR "old resident*" OR "old inhabitant*" OR senior* OR retired OR retiree* OR pension*  #5 #1 AND #2 AND #3 AND #4  Results: 146 |

Results together after duplicates (344) removed: 943
